# Evidence of positive selection associated with placental loss in tiger sharks

**DOI:** 10.1186/s12862-016-0696-y

**Published:** 2016-06-14

**Authors:** Dominic G. Swift, Luke T. Dunning, Javier Igea, Edward J. Brooks, Catherine S. Jones, Leslie R. Noble, Adam Ciezarek, Emily Humble, Vincent Savolainen

**Affiliations:** Department of Life Sciences, Imperial College London, Silwood Park Campus, Buckhurst Road, Berkshire, SL5 7PY UK; Department of Animal and Plant Sciences, University of Sheffield, Sheffield, S10 2TN UK; Department of Plant Sciences, University of Cambridge, Downing Street, Cambridge, CB2 3EQ UK; Shark Research & Conservation Program, Cape Eleuthera Institute, PO Box EL – 26029, Eleuthera, the Bahamas; Institute of Biological and Environmental Sciences, School of Biological Sciences, University of Aberdeen, Zoology Building, Tillydrone Avenue, Aberdeen, AB24 2TZ Scotland UK; Department of Animal Behaviour, University of Bielefeld, Postfach 100131, 33501 Bielefeld, Germany

**Keywords:** Reproduction, Viviparous, Placenta, Transcriptome, RNA-Seq, Positive selection, Elasmobranchs, Carcharhinids, *Galeocerdo*

## Abstract

**Background:**

All vertebrates initially feed their offspring using yolk reserves. In some live-bearing species these yolk reserves may be supplemented with extra nutrition via a placenta. Sharks belonging to the Carcharhinidae family are all live-bearing, and with the exception of the tiger shark (*Galeocerdo cuvier*), develop placental connections after exhausting yolk reserves. Phylogenetic relationships suggest the lack of placenta in tiger sharks is due to secondary loss. This represents a dramatic shift in reproductive strategy, and is likely to have left a molecular footprint of positive selection within the genome.

**Results:**

We sequenced the transcriptome of the tiger shark and eight other live-bearing shark species. From this data we constructed a time-calibrated phylogenetic tree estimating the tiger shark lineage diverged from the placental carcharhinids approximately 94 million years ago. Along the tiger shark lineage, we identified five genes exhibiting a signature of positive selection. Four of these genes have functions likely associated with brain development (*YWHAE* and *ARL6IP5*) and sexual reproduction (*VAMP4* and *TCTEX1D2*).

**Conclusions:**

Our results indicate the loss of placenta in tiger sharks may be associated with subsequent adaptive changes in brain development and sperm production.

**Electronic supplementary material:**

The online version of this article (doi:10.1186/s12862-016-0696-y) contains supplementary material, which is available to authorized users.

## Background

Aristotle was the first to record some animals give birth to live young (i.e. viviparity) whilst others lay eggs (i.e. oviparity) [1]. Viviparity may offer selective advantages to parents and offspring, such as enhanced survival of offspring, compensation for low fecundity, amplification of reproductive niches to reduce competition, exploitation of pelagic niches, colonisation of new habitats, and possibly increased energetic efficiency; disadvantages may include reduced fecundity, cost to the female, and risk of brood loss through maternal death [2]. Viviparity is thought to have first evolved over 350 million years ago (mya), and is an unprecedented example of convergent evolution having independently evolved at least 150 times in mammals, reptiles, amphibians, and fishes [3, 4]. Among these viviparous organisms there are differences in the method of foetal nutrition, with the supply of nutrients via yolk in eggs or yolk-sacs considered to be ancestral [1]. In fact, yolk-sac placentation is the most common type of placentation in vertebrates [5].

Oviparity also has its own benefits given it has been retained by the majority of vertebrates. Although viviparity has evolved many times, it creates post-fertilization opportunities for genomic conflicts absent in oviparous species [6, 7]. Furthermore, transitions in reproductive modes require numerous morphological, physiological, and behavioural adaptations. These can be associated with variation in geographic distribution and environmental conditions, as documented in reptiles [8, 9] and amphibians [10].

Sharks are considered the most enduring of fishes having survived the mass extinction events of the last 420 million years. Approximately 70 % of these species give birth to live young and approximately 30 % lay eggs [11, 12]. Sharks are among the first vertebrates to display viviparity [9]. They are also members of a lineage which is a sister group to all teleosts and tetrapods, thereby ideally placed for comparative studies of these taxa. To date, however, there have been few studies of sharks at genome level [13–15]; one of the reasons for this is the large size of shark genomes (up to 34 picograms per haploid genome; >30 Gb) [16] in comparison to bony fish models (e.g. zebrafish 1.4 Gb [17]).

During the early stages of development all viviparous sharks are nourished by a foetal yolk-sac [18]. Viviparous sharks also display a diversity of embryonic nourishment derived from maternally obtained nutrients, known as matrotrophic nutrition. Types of matrotrophic nutrition in sharks include mucus produced by the uterus (mucoid histotrophy), supply of unfertilised eggs (oophagy), and direct exchange between maternal and foetal tissues via a placenta (placental viviparity). Placental connections develop in sharks when the empty yolk-sac morphs and attaches to the uterus wall [18]. In viviparous shark species that do not develop placental connections (i.e. non-placental), the empty yolk-sac is reabsorbed into the developing embryo [18].

Placenta are thought to have one evolutionary origin in sharks and are restricted to five families from the Carcharhiniformes order (i.e., Carcharhinidae, Sphyrnidae, Hemigaleidae, Leptochariidae, and Triakidae) [8]. Two of these families, Carcharhinidae and Triakidae, contain both placental and non-placental species, with the lack of placenta thought to be due to secondary loss [8]. Additional nutrition via a placenta is suggested to increase the embryonic development rate of energetically expensive tissues such as the brain, and there is evidence associating increased reproductive nutrition in sharks with larger brain sizes, relative to body mass [19].

Here we focused on one shark family, the carcharhinids (Order: Carcharhiniformes, Family: Carcharhinidae), which all display placental viviparity, apart from the tiger shark (*Galeocerdo cuvier*) [20]. As the sister groups to *Galeocerdo* are placental, it is most likely this lineage has undergone placental loss. Recent research has identified distinct embryonic nutrition displayed by tiger sharks where the egg case housing the embryo becomes filled with approximately one litre of energy-rich, yellowish fluid [21].

In this study, we reconstructed a phylogenetic relationship for the tiger shark, six other carcharhinid sharks, and two outgroup species. We used fossil data to produce a time-calibrated phylogeny to estimate when the tiger shark lineage diverged from the placental carcharhinids. We also aimed to identify orthologous genes among the transcriptomes of the nine species and determine if there is evidence of genes evolving under positive selection, and possibly associated with the loss of placenta in the tiger shark lineage. Based on the proposed link between embryonic nutrition and brain size, we hypothesised to detect in the tiger shark lineage evidence of positive selection in genes encoding proteins associated with brain development, as well as sexual reproduction.

## Results

### Sequencing

We sequenced the transcriptomes of six placental carcharhinids and the non-placental tiger shark. We also sequenced the transcriptomes of two additional shark species to serve as outgroups: the placental viviparous dusky smoothhound (*Mustelus canis insularis*) (Order: Carcharhiniformes, Family: Triakidae) and another species that does not develop a placenta, the sand tiger shark (*Carcharias taurus*) (Order: Lamniformes, Family: Odontaspididae) (Table 1). After quality control, Illumina sequencing of the white muscle transcriptomes of the nine species produced over 550 million filtered paired-end 100-base pair (bp) reads (Mean: 61,382,483 reads; Standard Deviation: 5,278,376 reads).

**Table 1:** Transcriptome statistics for the nine viviparous shark species sampled here

Species	Family	Order	Placental/Non-placental	Number of filtered reads	Number of transcripts	N50
Atlantic sharpnose shark (*Rhizoprionodon terraenovae*)	Carcharhinidae	Carcharhiniformes	Placental	60,513,987	88,870	1844
Blacknose shark (*Carcharhinus acronotus*)	Carcharhinidae	Carcharhiniformes	Placental	57,835,152	131,575	2201
Blue shark (*Prionace glauca*)	Carcharhinidae	Carcharhiniformes	Placental	65,764,260	96,764	1137
Bull shark (*Carcharhinus leucas*)	Carcharhinidae	Carcharhiniformes	Placental	60,513,987	91,122	1719
Caribbean reef shark (*Carcharhinus perezii*)	Carcharhinidae	Carcharhiniformes	Placental	62,012,857	118,363	2340
Dusky smoothhound (*Mustelus canis insularis*).	Triakidae	Carcharhiniformes	Placental	52,695,471	98,463	2026
Lemon shark (*Negaprion brevirostris*)	Carcharhinidae	Carcharhiniformes	Placental	62,258,228	70,506	1701
Sand tiger shark (*Carcharias taurus*)	Odontaspididae	Lamniformes	Non-placental	71,760,543	118,363	1687
Tiger shark (*Galeocerdo cuvier*)	Carcharhinidae	Carcharhiniformes	Non-placental	59,087,862	179,867	1858

### Transcriptome assembly and orthologue identification

We used Trinity (version 2013-05-08) to assemble species specific transcriptomes [22]. The mean number of transcripts was 109,709 (Standard Deviation: 31,801) and the mean N50 value was 1835 (Standard Deviation: 348). We then identified from the open reading frames (ORFs) of assembled transcripts 3,215 putative orthologous sequences using a reciprocal best-hit Blast search. High confidence alignments were generated for these orthologous sequences using multiple aligners. Alignments were subsequently filtered again to remove low confidence codon alignments, finally resulting in 1,197 orthologues for further analysis.

### Phylogenetic tree construction and positive selection analyses

The 1,197 orthologue alignments were concatenated (1,101,288 bp) and used to construct a phylogenetic tree (Additional file 1: Figure S1) using RAxML (version 8.0.0) [23]. The 1,197 orthologues were then analysed with CodeML, a program of PAML (version 4.7) [24]. The CodeML one ratio model (M0) found no orthologue exhibiting signatures of positive selection across the entire sequence. Therefore, we used an additional test, a comparison between the neutral model 7 (M7) and the non-neutral model 8 (M8) to identify specific regions of genes that may be evolving under positive selection. Sequences with significant M8:M7 likelihood ratios provide evidence of positive selection; therefore these sequences were further analysed using the Bayes Empirical Bayes (BEB) method to identify specific codons which may be under positive selection [25]. Of the 1,197 orthologues across nine species analysed by CodeML and BEB, 95 orthologues (Additional file 2: Table S1) were found to have specific codon sites showing signatures of positive selection (Benjamini-Hochberg corrected *p* value < 0.05).

**Fig. 1:**
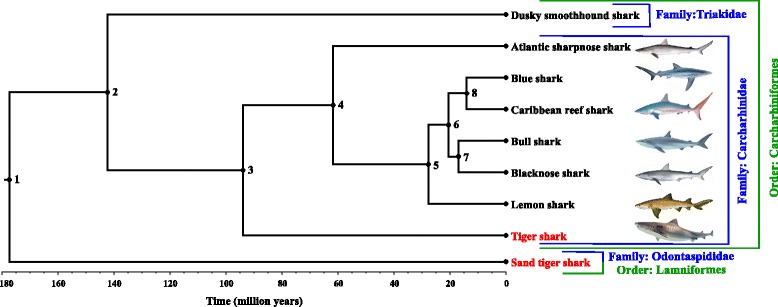
Time-calibrated phylogenetic tree of sharks. Based on analyses of 1,102 genes (1,007,817 bp per species). Species are named along with the orders and families they belong to the non-placental species are shown in red. Each node was annotated with inferred posterior mean times and 95 % highest posterior density credibility intervals in million years. Node 1: 177 and 170–184. Node 2: 142 and 130–163. Node 3: 94 and 59–130. Node 4: 62 and 35–88. Node 5: 28 and 20–37. Node 6: 21 and 15–27. Node 7: 17 and 11–22. Node 8: 14 and 10–17. Each node is supported with a bootstrap value of 100 %

Another phylogenetic tree was reconstructed, but this time only using orthologues not evolving under positive selection (Fig. 1). Using this second tree, and re-mapping all orthologues onto the tree, signatures of positive selection were identified at specific codon sites within five genes (*NARS2*, *VAMP4*, *TCTEX1D2*, *YWHAE*, and *ARL6IP*) of the non-placental tiger shark lineage using the branch-site test and BEB method (Benjamini-Hochberg corrected *p* value < 0.05). Two of these genes are associated with sexual reproduction (*VAMP4* and *TCTEX1D2*) and two are associated with brain development (*YWHAE* and *ARL6IP5*). For *NARS2*, we could not find sufficient information that could link positive selection in this gene to the loss of placental viviparity.

Note that the topologies of both phylogenetic trees (i.e. using all orthologues or the subset not evolving under positive selection) were identical and well supported, all nodes receiving 100 % bootstrap support. Data from fossil teeth were placed on five nodes (Table 2; Fig. 1) to obtain a time-calibrated phylogenetic tree using the MCMCTree function of PAML [24, 26–32]; four independent runs of MCMCTree produced the same dates for those nodes.

**Table 2:** Fossil calibration used for calibrating the shark phylogeny. Minimum and upper bound fossils with estimated ages and references for nodes 1, 2, 3, 5 and 8

Calibration Point	Fossil (minimum age)	Lower Bound Fossil Age (mya)	Reference	Fossil (maximum age)	Upper Bound Fossil Age (mya)	Reference
Lamniformes – Carcharhiniformes (Node 1)	*Paleoscyllium tenuidens*	169.6	Underwood & Ward, 2004 [31]	*Paracestracion*	183.4	Delsate & Lepage, 1990 [28]
Triakidae – Carcharhinidae (Node 2)	Carcharhiniformes Fossil	132.9	Underwood et al. 1999 [30]	*Paleoscyllium tenuidens*	169.6	Underwood & Ward, 2004 [31]
*Galeocerdo* – Carcharhinidae (Node 3)	*Galeocerdo latidens*	47.8	Noubhani & Cappetta, 1997 [29]	Carcharhiniformes Fossil	132.9	Underwood et al. 1999 [30]
*Negaprion* – *Carcharhinus*/*Prionace* (Node 5)	*Aprionodon acutidens*	16.0	Probst, 1879 [26]	*Galeocerdo latidens*	47.8	Noubhani & Cappetta, 1997 [29]
*Prionace – Carcharhinus perezii* (Node 8)	*Squalus glauca*	3.6	Landini, 1977 [27]	*Aprionodon acutidens*	16.0	Probst, 1879 [26]

## Discussion

### Phylogenetic relationships

Using 1,007,817 bp of transcriptome data per species we reconstructed phylogenetic relationships for the species sampled. We included the dusky smoothhound and sand tiger shark as outgroups; the placental dusky smoothhound belongs to the only other shark family with both placental and non-placental species (Triakidae), whereas the sand tiger shark is one of many non-placental species making up a sister order to the Carcharhiniformes. The Carcharhiniformes and Lamniformes appear to have diverged before the evolution of placenta in sharks.

The resulting phylogenetic relationships are largely consistent with others recently published [33], except for placement of the Caribbean reef shark. In the Sorenson et al. [33] phylogenetic tree the Caribbean reef shark is positioned as a sister group to a clade including the bull and blacknose sharks, with the blue shark as an outgroup to the three species. In our phylogenetic trees, the blacknose and bull sharks are grouped into one clade and the blue and Caribbean reef sharks are grouped into another clade (Fig. 1). We believe our topology is more robust given the larger number of characters used (i.e. over a thousand nuclear genes versus one nuclear and four mitochondrial genes by Sorenson et al.), although here with a limited taxon sampling. We estimated the tiger shark lineage diverged from the placental carcharhinids approximately 94 mya (59-130 mya, 95 % highest posterior density credibility interval (HPD CI)), whereas the estimate of Sorenson et al. [33] was 80 mya (65-95 mya, HPD CI) (Table 3). It should be noted, however, that Sorenson et al. employed a much denser taxonomic sampling (268 species compared with nine species in our study) that contributed to shorter branch lengths, smaller HPD CIs, and also allowed for additional fossil calibrations. The relatively old ages of our outgroups and the long branch lengths at the base of our phylogenetic trees likely contribute to the larger HPD CI for the divergence of the tiger shark and placental carcharhinid lineages compared to Sorenson et al. In addition, cartilaginous skeletons contribute to the paucity of shark fossils and consequently teeth are one of the most abundant shark fossil types [34, 35]. Identifying species based on fossilised teeth is more difficult than identifying based on complete fossils as sharks exhibit diversity in dental morphology across sexes, life history stages, and even positions within the jaw [36]. Thus there is a level of uncertainty associated with the fossil data used to calibrate the phylogeny.

**Table 3:** Inferred divergence times for the Carcharhinidae – *Galeocerdo* node in shark phylogenetic trees. Mean age and 95 % highest posterior density credibility intervals (HPD CI) are provided for this study and Sorenson et al. [33]

Node	This Study		Sorenson et al. 2014 [33]	
	Mean Age (mya)	95 % HPD CI (mya)	Mean Age (mya)	95 % HPD CI (mya)
Carcharhinidae – *Galeocerdo*	94	59–130	80	65–95

### Genes evolving under positive selection in the tiger shark

#### Positive selection on genes linked to sexual reproduction

Tiger sharks have a gestation period of approximately 15-16 months, several months longer than many placental carcharhinids, and follow a triennial reproductive cycle, with an estimated one year of sexual inactivity [37]. As male tiger sharks generally reproduce annually, changes to female reproductive behaviour may have led to an increase in sperm competition and post-copulatory sexual selection.

Here, we detected the signature of positive selection in the *TCTEX1D2* and *VAMP4* genes. *TCTEX1D2* encodes a dynein-2 light chain protein required for cilia function and found in the flagellum of sperm in a variety of taxa, including humans, mice, teleosts and sea urchins [38–40]. Sperm motility is reduced and spermatogenesis disrupted in mice lacking *TCTEX1D2* as a result of increased apoptosis in male germ cells [41]. Deletion of *VAMP4* causes a significant increase in sperm head abnormalities in mice, resulting from aberrant acrosome formation [42]. This has implications for sperm morphology and hydrodynamics, therefore positive selection in *TCTEX1D2* and *VAMP4* may alter sperm count and motility, which could be a response to changes in sperm competition in tiger sharks induced by the loss of placenta.

It is assumed when there is strong sperm competition an individual can gain a competitive advantage by increasing sperm production [43]; however, there is a trade-off between sperm size and number [71]. Sharks display extensive variation in sperm morphology which is thought to demonstrate variation in the intensity of post-copulatory sexual selection [44–46]. Tanaka et al. [44] showed tiger sharks have the shortest sperm flagella length and total length of 27 sharks across seven orders. Therefore, evidence of positive selection in *TCTEX1D2* and *VAMP4* in tiger sharks could reflect the evolution of an increased sperm count and shorter sperm in response to increased sperm competition and post-copulatory sexual selection.

#### Positive selection on genes linked to brain development

Signatures of positive selection were detected in *ARL6IP5*, a transmembrane protein inhibiting *EAAC1*, the latter being a transporter of the excitatory neurotransmitter glutamate [47]. *EAAC1* is associated with neuron development in both vertebrates and invertebrates [48, 49]. When deprived of oxygen, levels of glutamate in neurons increase, causing neuronal death and potentially brain damage [50]. Therefore, the ability to maintain glutamate levels below damaging thresholds may have enabled the exploitation of marginal habitats. Penetrating marginal habitats could be advantageous for these predators by allowing expansion into sub-optimal habitats where prey may take refuge [51]. Contemporary evidence for this is demonstrated by the near-global distribution of tiger sharks in coastal and pelagic habitats around the world, as well as their diverse diet [52]. Tiger sharks also spend considerable time in shallow seagrass and neritic habitats [53, 54], where dissolved oxygen concentrations fluctuate diurnally due to high productivity and demand [55].

The brain is one of the most energetically expensive organs to develop and maintain, and non-placental sharks are proposed to generally have smaller brains relative to body mass, compared to placental species [19, 56]. Animals need to maintain a balance between maintenance of the brain and other organs when exposed to hypoxic conditions [57]. There is evidence of intra and interspecific variation in brain size of fishes exposed to different concentrations of dissolved oxygen, with larger brains evident in species inhabiting well-oxygenated waters [57–59].

Signatures of positive selection were also detected in *YWHAE*, a member of the 14-3-3 protein family [60]. These proteins are expressed ubiquitously, particularly in the brain, and are highly conserved across animals [60, 61]. The 14-3-3 proteins are vital for differentiation of neurons in *Drosophila*, while mice lacking *YWHAE* have restricted brain development and neuronal migration [60, 62]. In humans, *YWHAE* is absent in sufferers of Miller-Dieker syndrome, which is characterised by severe mental disability [60]. This suggests *YWHAE* is vital for brain development in humans and other animals. Positive selection in *YWHAE* could thus reflect a reduction in brain size in response to hypoxia experienced in sub-optimal habitats occupied by tiger sharks.

Positive selection in the *TCTEX1D2*, *VAMP4*, *ARL6IP5*, and *YWHAE* genes and their functional associations suggest they may have played a role in adaptation following the loss of placenta in tiger sharks. Noteworthy, changes in gene expression could have also been involved; however, as the time since species divergence increases, so do gene expression differences [74]. Given the tiger shark and placental carcharhinids diverged c. 94 mya, the magnitude of gene expression changes is considerable, likely erasing any potential signal of gene expression changes associated with the loss of placenta. Furthermore, controlling the variety of factors (e.g. environment, sex, life history stage) potentially influencing gene expression would require a completely different sampling design to the one we employ here. Investigations of gene expression would also require uterus or yolk-sac tissue which are difficult to sample in a non-lethal way; hence, we sampled white muscle tissue instead. Furthermore, positive selection is intermittent in nature and signatures of it can be lost over time due to recombination and accumulating neutral substitutions [63, 64], thus some signatures of positive selection in other genes may no longer be detectable.

Previous studies have shown positive selection can act strongly on genes that greatly affect an individual’s fitness (e.g. sexual reproduction and sensory perception) [65, 66]. Thus, there could be additional factors explaining the signatures of positive selection exhibited in these four genes in the tiger shark lineage. We have here only one point of comparison, these genes might affect other traits of the tiger shark and may not be linked simply to the loss of placenta. Their functional associations, however, suggest these genes are good candidates for further study using additional genomic techniques.

Other studies have used genomics to test for signatures of positive selection associated with placental evolution in mammals [67, 68]. Evidence of positive selection in this group was found across 1,254 genes by Crosley et al. (2013) and in approximately 300 genes by Elliot & Crespi (2015). Signatures of positive selection were detected in a greater number of genes in these studies compared with our study; however, both Crosley et al. and Elliot & Crespi used a larger set of closely related species, and therefore looked at more genes than us, i.e. 16,578 genes in Elliot & Crespi (2013) and approximately 18,000 genes in Crosley et al. (2015) versus 1,197 genes here. In addition, Crosley et al. and Elliot & Crespi tested for positive selection along multiple lineages, whereas we tested for positive selection along the tiger shark lineage only. Interestingly, signatures of positive selection were also detected in genes associated with brain development and sexual reproduction in Elliot & Crespi and Crosley et al., respectively [67, 68], suggesting similar adaptations associated with placentation may have occurred in both mammals and sharks.

We were limited in the outgroups we could sample. We included as an outgroup one placental species (i.e. dusky smoothhound) from the other shark family (i.e. Triakidae) in which species may have also lost placenta. Unfortunately, we could not obtain samples from non-placental houndshark species, and so could not compare signatures of positive selection in genes along two independent lineages where placenta may have been lost. Also, we were unable to sample additional carcharhiniform species, but had lamniforms instead (which do not develop placenta). We identified more orthologous genes in the sand tiger transcriptome compared with other potential outgroup species, and so we used it as the second outgroup taxon.

Finally, another caveat of our study is the sampling of a single individual for each species during a short time frame. Consequently, the sequence data for each species lack individual, gender, ontogenetic, and temporal variation.

## Conclusions

We suggest the tiger shark lineage split from the placental carcharhinids approximately 94 mya. We also propose at least four genes associated with brain development and sperm production have been evolving under positive selection in the tiger shark lineage, potentially reflecting adaptation following placental loss. Future work should utilise supplementary genomic techniques to investigate similar changes in reproductive traits of additional shark species.

## Methods

### Sample collection

In total, one individual from six carcharhinid species were sampled, the non-placental tiger shark and five placental carcharhinids: Atlantic sharpnose (*Rhizoprionodon terraenovae*), blacknose (*Carcharhinus acronotus*), bull (*Carcharhinus leucas*), Caribbean reef (*Carcharhinus perezii*), and lemon (*Negaprion brevirostris*) sharks. A placental dusky smoothhound individual was also sampled. Collection of white muscle samples was carried out off the coast of Eleuthera, the Bahamas (N 24° 50’ 05”: W 076° 19’ 32”) in a two-week period covering January and February 2014. The seven shark species were caught using 400 m stationary longlines with 30 to 33 non-offset, 16/0 circular hooks (Mustad, Gjövik, Norway). Hooks were spaced five metres apart and were baited with Atlantic bonito (*Sarda sarda*). The longlines were left in the water for approximately 90 min. White muscle samples were collected using a biopsy punch from an area adjacent to the dorsal fin. Samples were immediately placed in RNAlater (Sigma, St. Louis, MO, USA) and stored at 4 °C for 24 h, before storing at −20 °C. Samples were stored in an icebox for 16 h during transport back to the UK.

### Ethics approval

Sample collection was carried out under Cape Eleuthera Institute animal care protocols developed within the guidelines of the Association for the Study of Animal Behaviour and the Animal Behaviour Society [69]. All sample collection activities were approved by The Bahamas Department of Marine Resources under research permits MAF/FIS/17 and MAF/FIS/34.

### RNA extraction and purification

Muscle tissue samples were removed from RNAlater and individually left to dry in a Petri dish for five minutes before being cut up finely with a scalpel and individually homogenised in 300 μl of Buffer RLT (Qiagen, Hilden, Germany) using a PowerGen 120 Homogeniser (Fisher Scientific, Loughborough, UK). Total RNA was extracted using the RNeasy fibrous tissue mini kit (Qiagen, Hilden, Germany), following the manufacturers protocol.

RNA quantity was assessed using a NanoDrop 2000 spectrophotometer (NanoDrop Technologies, Wilmington, DE, USA) and RNA quality assessed by electrophoresis on 1 % TAE-agarose gels. RNA samples were subjected to a cleaning and concentration phase using the RNeasy cleanup kit (Qiagen, Hilden, Germany), before being further assessed for quality and quantity using a Bioanalyzer (Agilent Technologies, Palo Alto, CA, USA). At least 500 ng of RNA with an RNA integrity number (RIN) of 6.0 or above, were stored at -80 °C prior to being sent for sequencing. In addition to the seven species sampled, filtered paired-end reads from the white muscle transcriptomes of the placental blue shark (*Prionace glauca*) (Order: Carcharhiniformes, Family: Carcharhinidae) and sand tiger shark were procured. For each species, a single individual was sampled and a transcriptome sequenced. The reads were generated following the same methods used for the other species in this study.

### Sequencing, quality control, and *de novo* assembly

A normalised cDNA library was synthesised and sequenced for each species by BGI Tech Solutions (Hong Kong) using the Illumina TruSeq kit (Illumina, San Diego, CA, USA). These libraries were later sequenced using RNA-Seq and an Illumina HiSeq 2000 system following standard protocol. cDNA libraries were normalised in order to maximise transcript coverage and to sequence as much of the complete transcriptomes as possible. BGI Tech carried out an initial round of quality control, trimming raw reads, primers and adaptor sequences, as well as removing low quality reads (Phred quality < 20). FastQC (version 0.10.1) further assessed the filtered reads before assembly [70]. The transcriptomes were *de novo* assembled using Trinity (version 2013-05-08) with default parameters [22].

### Identification and alignment of orthologous sequences

The transcriptomes of all nine species were clustered separately using the Uclust function of Usearch (version 7.0.1090) with a similarity threshold of 98 % to breakdown and remove putative splice variants [71]. The Trinity ORF predictor was employed using TransDecoder (version 2013-05-08) to calculate the longest and most probable translated region for each sequence and to remove multiple ORF sequences. A custom Perl script was used to remove all stop codons from the end of ORF sequences.

Pairwise reciprocal Blast searches were employed using Blastn (version 2.2.29) to identify putative orthologous sequences shared between each pair of species [72]. Pairwise putative orthologues were then collated using R (version 3.1.1) in order to find sequences shared between all nine species. The multiple sequence alignment program M-Coffee (version 11.00.9103146) was used to align orthologous sequences between all species using Mafft, Muscle, T-Coffee, and Kalign methods [73]. Gblocks was also used to remove poorly aligned and divergent regions to further reduce alignment errors and gaps [74]. The Gblocks parameters used were: minimum of seven sequences for a conserved position, minimum of seven sequences for a flank position, maximum of six contiguous non-conserved positions, minimum block length of nine, and 50 % or more of sequences with a gap were treated as a gap position. M-Coffee translated nucleotide sequences into amino acid sequences, which were aligned for all species and then back-translated to nucleotide sequences. Alignments were then trimmed and graded. Only alignments with quality grades of nine, the highest score, were retained for further analysis.

### Phylogenetic tree construction and positive selection analyses

The aligned orthologues were concatenated and used to construct a phylogenetic tree using RAxML (version 8.0.0) [23]. We utilised the nucleotide substitution model GTRGAMMA, as determined by jModelTest (version 2.1.4) using the lowest value of the Akaike Information Criterion [75, 76]. Bootstrap values were calculated using 1,000 replicates. CodeML calculated the number of substitutions which alter the amino acid sequence (nonsynonymous (*dN* )) and the number of substitutions which do not alter the amino acid sequence (synonymous (*dS*)) [24]. The ratio of these substitutions can be used to detect genes exhibiting signatures of positive selection. Positive selection, the favouring of distinct phenotypes, can be indicated by a *dN* / *dS* ratio (ω) > 1. ω < 1 is indicative of negative selection, the removal of deleterious alleles, and ω = 1 can indicate neutral selection, drift of alleles not affecting an individual’s ability to pass on their genes [77]. It is suggested positive selection can only be detected if the average ω across all codon sites, calculated by the one ratio model (M0), is greater than 1. This is conservative, however, considering most codon sites will be highly conserved to maintain protein structure and function [78]. Also, estimates of ω can be artificially decreased due to partial sequences produced by high-throughput sequencing technologies [79].

A maximum-likelihood site test based on a comparison between the neutral model 7 (M7) and the non-neutral model 8 (M8) was therefore employed. M7 assumes a β distribution of ω between 0 and 1, not allowing ω > 1 at any sites [80]. M8 also assumes a β distribution of ω but allows an additional category of sites were ω > 1 [80]. A CodeML likelihood ratio test (LRT) was used to test if M8 fits the data significantly better than M7. The natural log likelihood (lnL) values of M7 were contrasted with those of M8. The lnL ratios were then compared to a chi-squared distribution with two degrees of freedom. False positive results are possible when implementing positive selection analyses on a genomic scale, therefore the Benjamini-Hochberg false discovery rate (FDR) correction was applied to all *p* values [81, 82]. The BEB method was employed for sequences with significant M8:M7 likelihood ratios and used to identify codon sites exhibiting signatures of positive selection [25]. Codon sites were considered to be showing evidence of positive selection if the probability of ω > 1 was more than 95 %.

We concatenated the 1,102 orthologues not showing signatures of positive selection for each species to construct a phylogeny in RAxML [23]. We used the nucleotide substitution model GTRGAMMA, as determined by jModelTest [76]. Bootstrap values for this phylogeny were calculated using 1,000 replicates. Fossil data were used to produce a time-calibrated phylogenetic tree (Table 2). This was accomplished using four independent runs of the MCMCTree function of PAML with 50,000 iterations, a burn in of 10,000 iterations, a sample frequency of three, an independent molecular clock, and the nucleotide substitution model HKY85, determined using jModelTest [76].

The branch-site test was utilised to detect signatures of positive selection at specific codon sites in the tiger shark lineage. The branch-site test is considered more powerful than the site test as signals of positive selection are not averaged over all branches of the phylogeny [78]. For the branch-site test, an alternative hypothesis was contrasted to the null hypothesis using an LRT where the lnL values were compared to a chi-squared distribution with one degree of freedom. Estimates of ω were not determined using M0 as this model has been shown to be unreliable when detecting positive selection in specific branches [78, 83]. The branch-site test has also been known to experience convergence problems when calculating likelihoods, leading to artificial lnL ratios [78]; thus, three independent runs of this model were performed for both the alternative and null hypotheses, with the highest lnL values kept to calculate the lnL ratios. The Benjamini-Hochberg FDR correction was applied to all *p* values [81]. The BEB method was employed to identify codon sites exhibiting signatures of positive selection [25]. Codon sites were considered to be showing evidence of positive selection if the probability of ω > 1 was more than 95 %.

## Abbreviations

BEB, Bayes Empirical Bayes; bp, base pairs; d_N_, number of nonsynonymous substitutions; d_S_, number of synonymous substitutions; FDR, false discovery rate; HPD CI, 95 % highest posterior density credibility interval; lnL, natural log likelihood; LRT, likelihood ratio test; M0, one ratio model; M7, neutral model 7; M8, non-neutral model 8; mya, million years ago; ORFs, open reading frames; RIN, RNA integrity number; TAE, Tris base, acetic acid and EDTA; ω, d_N_/d_S_ ratio

## Additional files

Additional file 1: Figure S1. Phylogenetic tree of sharks. Based on analyses of 1,197 genes (1,101,288 bp per species). Species are named along with the orders and families they belong to. ‘Lam.’ refers to Lamniformes order and ‘Tri.’ refers to Triakidae family. The non-placental species are shown in red. Each node is supported with a bootstrap value of 100 %. (PDF 25 kb) Additional file 2: Table S1. Orthologues Under Positive Selection Across All Species. The 95 orthologues exhibiting signatures of positive selection across all species based on the CodeML maximum-likelihood site test and Bayes Empirical Bayes method. (XLSX 29 kb)
