# Supplementary figures and images for: Evidence of positive selection associated with placental loss in tiger sharks
Source: BMC Evol Biol. 2016 Jun 14;16:126. doi: 10.1186/s12862-016-0696-y (PMC4906603; doi:10.1186/s12862-016-0696-y)

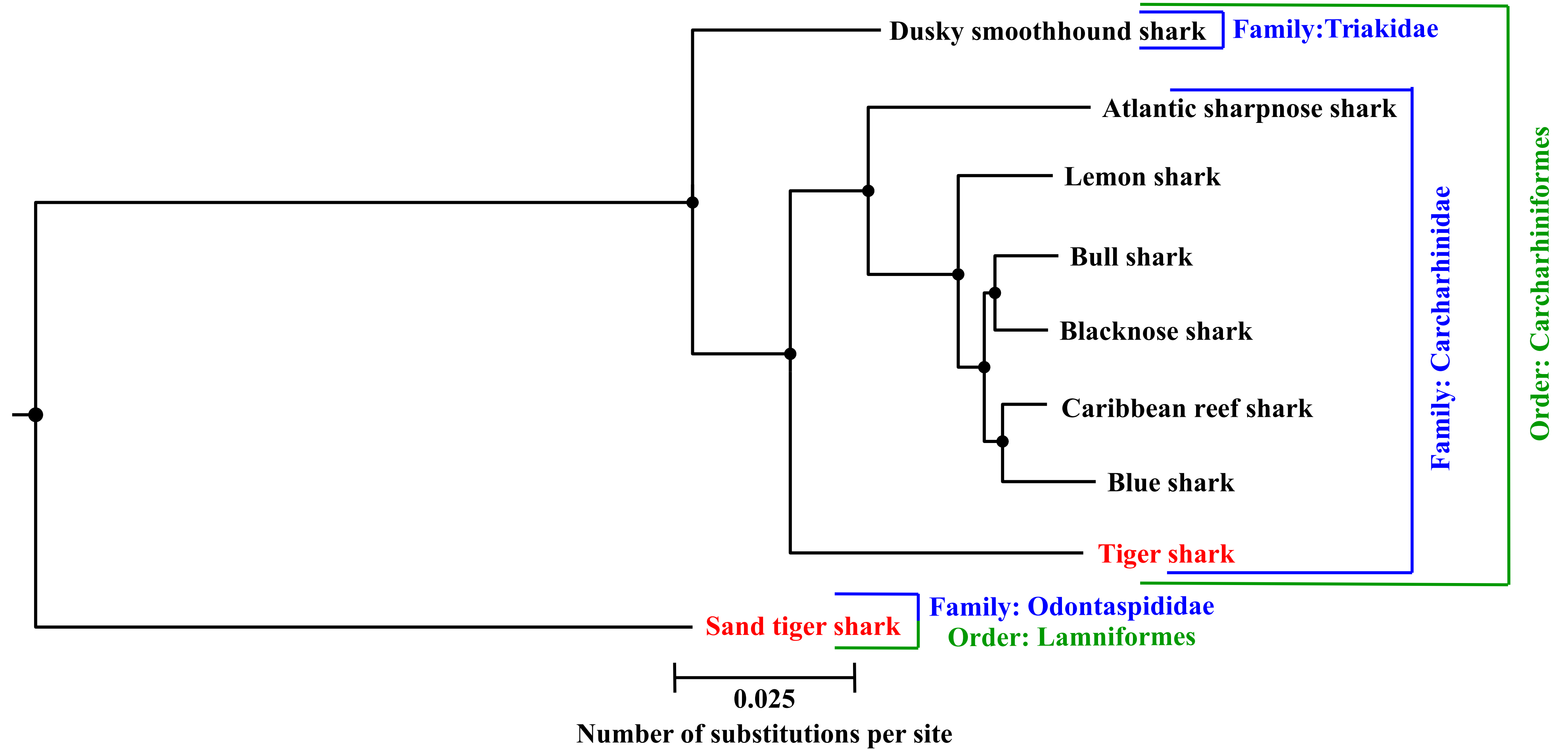

Supplement: Additional file 1: Figure S1. — Phylogenetic tree of sharks. Based on analyses of 1,197 genes (1,101,288 bp per species). Species are named along with the orders and families they belong to. ‘Lam.’ refers to Lamniformes order and ‘Tri.’ refers to Triakidae family. The non-placental species are shown in red. Each node is supported with a bootstrap value of 100 %. (PDF 25 kb) [file 12862_2016_696_MOESM1_ESM.pdf]
